# Malicious Code Variant Identification Based on Multiscale Feature Fusion CNNs

**DOI:** 10.1155/2021/1070586

**Published:** 2021-12-14

**Authors:** Shuo Wang, Jian Wang, Yafei Song, Song Li

**Affiliations:** College of Air and Missile Defense, Air Force Engineering University, Xi'an 710051, China

## Abstract

The increasing volume and types of malwares bring a great threat to network security. The malware binary detection with deep convolutional neural networks (CNNs) has been proved to be an effective method. However, the existing malware classification methods based on CNNs are unsatisfactory to this day because of their poor extraction ability, insufficient accuracy of malware classification, and high cost of detection time. To solve these problems, a novel approach, namely, multiscale feature fusion convolutional neural networks (MFFCs), was proposed to achieve an effective classification of malware based on malware visualization utilizing deep learning, which can defend against malware variants and confusing malwares. The approach firstly converts malware code binaries into grayscale images, and then, these images will be normalized in size by utilizing the MFFC model to identify malware families. Comparative experiments were carried out to verify the performance of the proposed method. The results indicate that the MFFC stands out among the recent advanced methods with an accuracy of 98.72% and an average cost of 5.34 milliseconds on the Malimg dataset. Our method can effectively identify malware and detect variants of malware families, which has excellent feature extraction capability and higher accuracy with lower detection time.

## 1. Introduction

Malware is a kind of malicious software that does harmful actions on computer systems, including viruses, worms, Trojan horses, and spyware [1]. According to the weekly report trends by the National Computer Network Emergency Response Technical Team/Coordination Center of China (known as CNCERT/CC), the number of hosts infected by network viruses in China was about 96,200 and the number of malicious programs transmitted in China was up to 69.724 million times only during one week [2]. With the increasing quantity and types of malwares, it becomes more and more difficult to detect these malwares, generating great challenges for network security. Therefore, quick and accurate methods to detect and classify malwares and their variants are highly desired in the professional field.

Feature vectors of malware represent the basic feature in malware detection. According to the different categories of malware feature vectors, malware analysis can be divided into dynamic analysis and static analysis. Static analysis, based on disassembling the malicious code, does not execute malicious code. The traditional methods of static analysis extract the attribute code, opcodes, and binary profiles of malware as a feature to identify sample malpractice. But code obfuscation frequently occurs in this approach. Differently, dynamic analysis is the practice of actually running an executable file and analyzing its behavior in a sandbox, simulator, and virtual machine. Some tools like ProcesMonitor or OllyDbg are used to monitor the application behavior through system calls [3]. Alazab et al. [4, 5] indicated that static analysis does better than dynamic analysis in the aspect of speed and effectiveness, because it can capture the information related to structural properties.

Traditional methods of malware detection are mainly based on malware feature analysis. However, the above methods do not identify malware variants. Nataraj et al. [6, 7] realized malware visualization by converting malware code binaries into malware grayscale images with the help of Conti et al. [8]. After malware visualization, malware images belonging to the same family are similar in vision, while malware images belonging to different families have a difference in vision. According to the characteristic of malware images, malware classification can be dealt with by computer vision.

Malware classification by visualization is proved to be faster and more accurate than traditional malware analysis methods. Also, these methods can resolve code obfuscation issues. In recent years, machine learning and deep learning are widely used to detect malware and malware classification based on malware visualization. Compared with other techniques of malware analysis, image texture analysis is proved to be a better way to the classification of the malware family variants. Currently, all the methods of malware classification based on malware images can be divided into two parts: extracting features from the malware images and realizing malware classification by classifiers, such as KNN (K-nearest neighbor) and softmax.

Machine learning based on malware visualization using malware dataset is adopted to train different machine learning classifiers. For example, Nataraj et al. [6, 7] proposed a method of malware classification that firstly extracted GIST features of malware grayscale images and then used the K-nearest neighbor to classify malware, obtaining a classification accuracy of 97.18% on the Malimg dataset. Kancherla and Mukkamala [9] reported a method that used 512 Gabor-based features, 22 wavelet-based features, and 6 intensity-based features as total features and SVM as a classifier to realize malware detection. In 2016, Nataraj and Manjunath provided a novel method named SPAM (signal processing for analyzing malware) that used images or signals to represent malware samples [10]. They first characterized malware by extracting the image- and signal-based features. Then, they used the GIST feature as a descriptor and nearest neighbor as a classifier to identify malware, achieving an accuracy of 97.40% on the Malimg dataset. In 2019, to reduce the computational time, Naeem et al. [11] proposed a LGMP feature description, which contains both local and global feature of malware images with a KNN classifier to detect malware. They firstly utilized a D-SIFT descriptor extract local malware feature and then used a GIST descriptor to extract global malware feature. Finally, a LGMP description was generated by combining local and global feature vectors. These results indicated that their method had a lower response time and a better performance on malware classification.

In recent years, great breakthroughs have been achieved in deep learning in image processing and target detection and some excellent performances have been realized in these fields [12–14]. Thus, a large number of studies, related to malware classification based on malware visualization with the method of deep learning, have been carried out. For example, Kabanga and Kim [15] used a simple CNN structure, which consists of three convolution layers and two fully connected layers to identify malware, achieving good performance. Yue [16] came up with a method of a weighted softmax loss to optimize CNNs on malware classification. This method was realized by setting a new parameter *β*, which can control the scaling of the weighted loss. It was proved an effective method by comparing the accuracy of the VGGNet [13] model and the VGGNet model with weighted softmax loss. Agarap [17] dealt with malware classification by combining deep learning and machine learning. They made use of deep learning, such as CNNs, GRU, and MLP, to extract features of malware images and then used SVM, a machine learning classifier, as the model classifier. However, the dimension of feature vectors extracted by deep learning is huge, limiting the effect of SVM, which resulted in low accuracy of 84.92%. The above models failed to deal with the imbalance dataset.

Cui et al. [18, 19] were devoted to deal with the data imbalance among different malware families by swarm intelligence algorithm, which in 2018 is the bat algorithm (DRBA) and in 2019 is NSGA-II. The accuracy of the training model was taken as an objective function. In the model training process, a sample of each malware family was resampled according to the weight, which is optimized by a swarm intelligence algorithm for each epoch. After making sure the best sample set, they trained a CNN model on this dataset to identify malware. Compared with the machine learning method of GIST + KNN, GIST + SVM, GLCM + SVM, and GLCM + KNN, their method generated a higher accuracy. In addition, there are other methods [20–22] dealing with the problem of malware family imbalance by a cost-sensitive approach.

Overall, most of the methods [23, 24] in malware classification by malware visualization are faced with the drawback of costing high in extracting features of malware images, such as GIST, GLCM, and LBP, leading to low efficiency. To reduce the cost of feature extraction and enhance the capability of feature extraction, a malware family classification approach with higher accuracy and lower detection time is highly required, which has more efficient feature extraction.

In this study, we propose a novel method, called MFFC (multiscale feature fusion on convolutional neural networks), to identify malware and detect variants of malware families, which have excellent feature extraction capability and higher accuracy and faster detection time.

The remainder of this study is structured in the following manner: Section 2 explains the approach, MFFC, which we have proposed in detail.  Section 3 introduces the datasets and statistical measures of the experiments.  Section 4 verifies the performance of our method.  Section 5 is devoted to analyzing the result of the comparative experiments.  Section 6 provides the concluding remarks and future direction.

## 2. Methods

**Figure 1:**
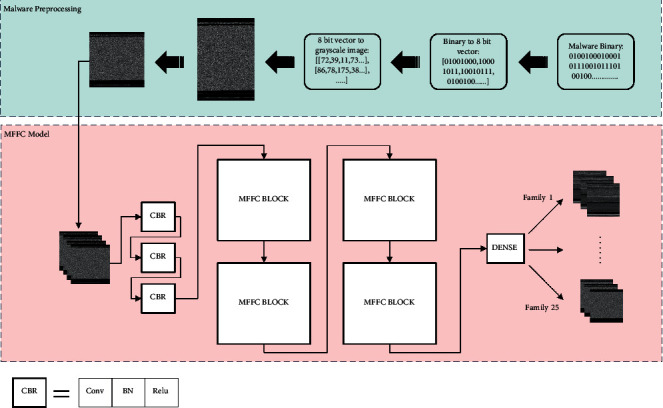
The basic structure of the MFFC algorithm.

MFFC mainly consists of two parts: malware preprocessing including malware visualization and malware image size normalization, and the MFFC model construction. The basic structure of the MFFC algorithm is depicted in Figure 1.

### 2.1. Malware Preprocessing

#### 2.1.1. Malware Visualization

**Figure 2:**
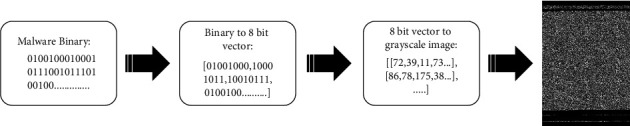
Visualizing malware as a grayscale image.

In 2010, Conti et al. proposed a method of mapping binary files into grayscale images by using multidimensional information theory to classify regions [8]. In 2011, Nataraj et al. [6] took the lead in applying the ideas of Conti et al. to the study of malware code. The method of malware visualization is as follows: the malware binary file is transformed into a vector of 8 bit unsigned integers (with a range of 0–255). The transformed vector is reconstructed into a 2D array according to different file sizes, and the 2D array is drawn as a grayscale image. The visualization processing of malware is shown in Figure 2. Based on [6, 7], different image widths should be set as in Table 1 according to different file sizes.

**Figure 3:**
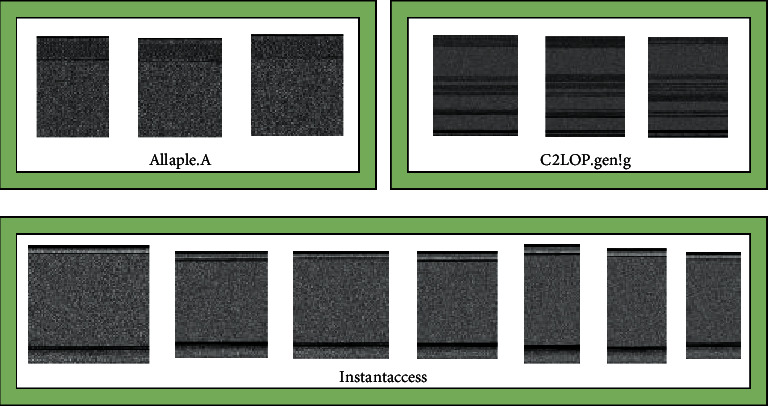
Samples of different malware family grayscale images.

The grayscale images of different malicious families are shown in Figure 3. It can be observed that although the malware grayscale image size and its ratio of length and width in the same family are different, and there are still similarities in vision, while the grayscale image samples of malware in different families are different in vision. That makes it possible to realize malware classification based on features of malware images.

According to the visual similarity of malware images, malware classification problem can be turned into computer vision problems.

#### 2.1.2. Malware Image Size Normalization

In the classical convolutional neural network, the size of the weight matrix that belongs to the full connection layer is fixed, so the number of neurons that is input to the full connection layer must be fixed. It means that the feature size after the convolution and pooling operation must be consistent before the full connection layer. If the size of the input image is different, the output feature size will also be different after the convolution and pooling operation, which will lead to the failure of the full connection layer. Thus, the images that feed into the neural network must be of the same size. However, the method of malware visualization determines that the size and ratio of malware images are different from each other. Thus, it is necessary to normalize the image size of sample images in the dataset.

In our study, malware images were reshaped to the fixed square sizes (e.g., 32*∗*32 and 64*∗*64). Only malware images that had already been normalized in image size could be fed into the CNNs for training. Malware image size normalization has the advantage that the dimensionality of the image can be effectively reduced, which does contribute to model training. Meanwhile, that will inevitably cause the loss of feature information during the process of dimensionality reduction.

**Figure 4:**
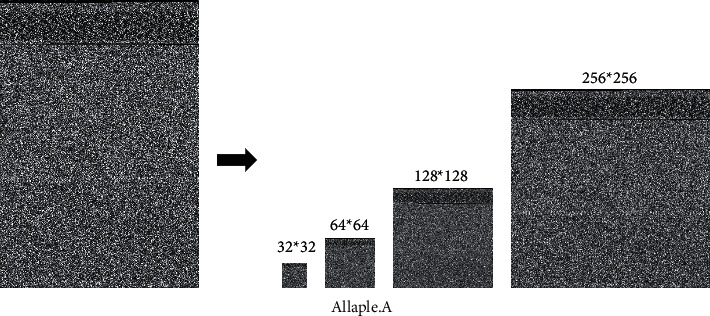
Visualizing malware as a grayscale image.

In Figure 4, we can see one of the grayscale images in the malware family named Allaple.A, which original size is 370*∗*256. It is resized to the different scales of 32*∗*32, 64*∗*64, 128*∗*128, and 256*∗*256. Obviously, the pivotal features of malware image can be preserved after image scaling.

### 2.2. MFFC Model

The MFFC model is shown in the pink part of Figure 1. The malware images after malware preprocessing will be fed into the MFFC model for training. In the MFFC model, there are three CBR layers, four MFFC blocks, and a dense layer with activation of softmax acting as a classifier, containing 25 classes.

**Figure 5:**
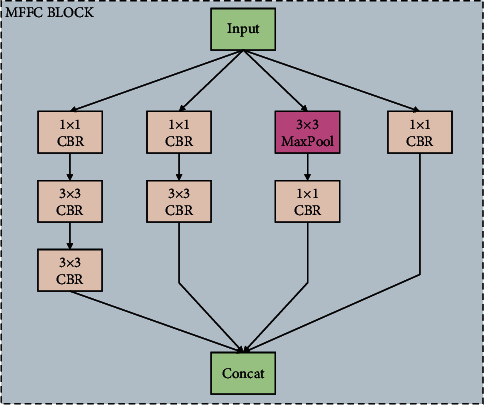
The structure of the MFFC block.

In order to enhance the ability of feature extraction of the MFFC model, the MFFC block is designed. The MFFC block is a block that is devoted to extract multiscale features of malware images. The structure of the MFFC block is shown in Figure 5.

In the MFFC block, there are four branches. Branches from right to left, respectively, are branch I, II, III, and IV. Branch I to IV will, respectively, generate feature vectors of C1, C2, C3, and C4. Finally, feature vectors C1 to C4 will be concatenated to get the final output. In the MFFC block, 1 × 1 CBR layers are used to reduce dimension, which can make the parameters decrease. In branch IV, two 3 × 3 CBR layers are to get a bigger receptive field.

## 3. Datasets and Statistical Measures

### 3.1. Datasets and Experimental Setup

**Figure 6:**
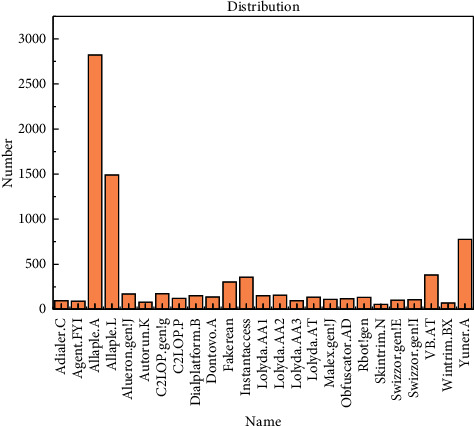
The distribution of the Malimg malware dataset.

All the experiments are evaluated on the Malimg malware dataset [6]. Malimg malware dataset consists of 25 malware families that have 9,435 malwares in total.  Figure 6 shows the distribution of samples in each malware family. We used 90% of the dataset for training and 10% of the dataset for testing.

### 3.2. Statistical Measures

For evaluating the performance of the approaches, four evaluation metrics, such as accuracy, precision, recall, and F1 score, are considered. The abovementioned evaluation metrics have been generally applied to related research studies for better assessments of various approaches [25–27]:

True positive (TP): it means that the positive samples are correctly detected as positive.
True negative (TN): it means that negative samples are correctly detected as negative.
False positive (FP): it means that negative samples are wrongly detected as positive.
False negative (FN): it means that a positive sample is wrongly detected as a negative.
Accuracy is defined as the ratio of correctly predicted outcomes to the sum of all predictions and is defined as follows:
  (1) $$\begin{array}{c} \text{accuracy}=\frac{\text{TP}+\text{TN}}{\text{TP}+\text{TN}+\text{FP}+\text{FN}}. \end{array}$$
Precision is the proportion of all the predicted samples that are correct (including positive and negative ones) in the total number of samples and is defined as follows:
  (2) $$\begin{array}{c} \text{precision}=\frac{\text{TP}}{\text{TP}+\text{FP}}. \end{array}$$
Recall is the proportion of the correct forecast positive to the total true positive and is defined as follows:
  (3) $$\begin{array}{c} \text{recall}=\frac{\text{TP}}{\text{TP}+\text{FN}}. \end{array}$$
F1 score is the weighted harmonic average of Precision and Recall and is defined as follows:

(4) $$\begin{array}{c} F1=2\times \frac{\text{precision}\times \text{recall}}{\text{Precision}+\text{recall}}. \end{array}$$

## 4. Results

To validate the effectiveness and efficiency of the proposed model (MFFC), we designed experiments as follows: (1) comparison of the performance with different malware image size, (2) performance of the MFFC algorithm, and (3) comparison of IMCFN performance over previously studied malware family classification techniques.

All experiments are conducted on 64 bit Windows Intel(R) Core(TM) i7-7700HQ CPU (2.80 GHz) with 16 GB RAM and NVIDIA GeForce GTX 1050 GPU (4 GB), based on python.

### 4.1. Comparison of the Performance with Different Malware Image Sizes

The input shape of the image to the CNN model is fixed limited by the full connection layer, but different input shapes of the malware image will get the different performance of the model. In order to obtain a more suitable size of the malware image, we normalize the malware images to different sizes as 32 × 32, 64 × 64, 128 × 128, and 256 × 256 to train the MFFC. The results are shown in Table 2.

When the input shape of the malware image is 256 × 256, the model achieves the highest accuracy of 98.72%, and its parameters are 1,104,041. When we predict a new malware sample, the model only costs 5.34 ms on average.

### 4.2. Performance of the MFFC Algorithm

**Figure 7:**
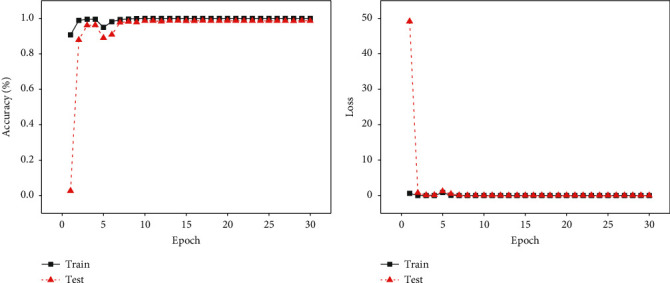
The details of MFFC during the model training. (a) The accuracy rate changing with the epoch of MFFC during the model training. (b) The loss changing with the epoch of MFFC during the model training.

Figure 7 shows the performance changes with an epoch of the train set and test set in the process of model training, where Figure 7(a) is the curve of accuracy rate changing with epoch and Figure 7(b) is the curve of loss changing with epoch. The black line represents the train set, while the red line is the test set. We can see that model has converged when the epoch is 7. After training and testing, we achieve an accuracy of 98.72% and a loss of 0.0517 for MFFC.

**Figure 8:**
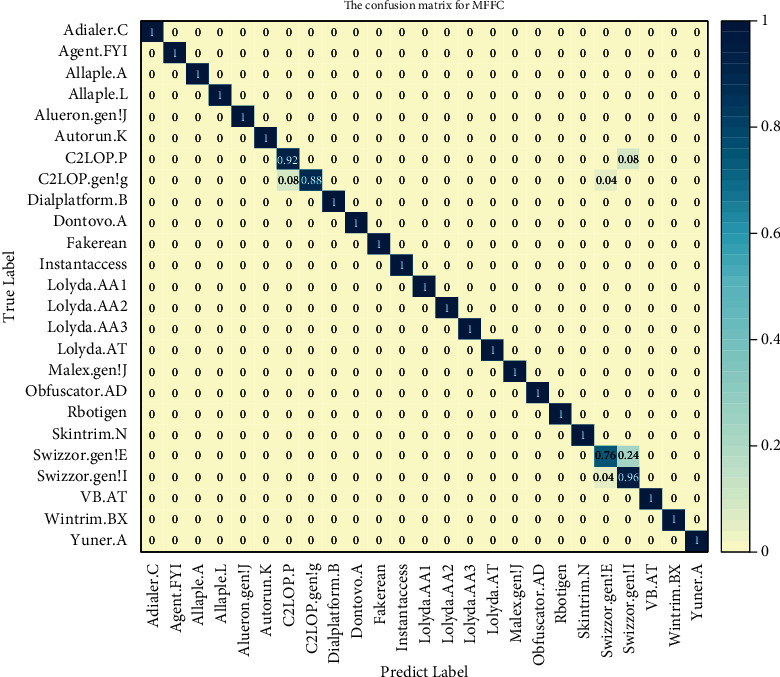
The confusion matrix for MFFC.

In order to clearly observe the classification details of the model, the confusion matrix for MFFC is plotted, as shown in Figure 8. The value of the leading diagonal in the confusion matrix represents the true-positive rate of malware family classification, and the other values mean the false-negative rate of malware family classification.

**Figure 9:**
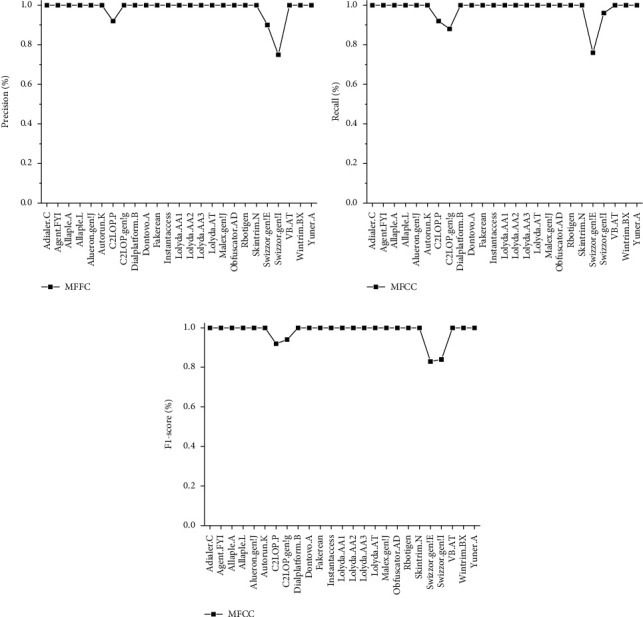
The performance of MFFC in 25 malware families. (a) The precision of MFFC in 25 malware families. (b) The recall of MFFC in 25 malware families. (c) The F1 score of MFFC in 25 malware families.

For this experiment, we obtain the precision of MFFC is 98.86%, while the recall is 98.72% and the F1 score is 98.73%. In Figure 9, the performance of MFFC in 25 malware families is shown.

### 4.3. Comparison with Existing Malware Classification

We compare the performance of MFFC with other approaches that are based on malware visualization, using machine learning or deep learning. All these approaches firstly convert the malware binaries into malware images, extracting features from the malware images, and then used machine learning classifiers (e.g., KNN and SVM) or deep learning classifiers (e.g., softmax) to classify the malware families.

Table 3 presents a comparative summary of the MFFC algorithm with previous malware classification algorithms that use the Malimg dataset to evaluate the experiment.

## 5. Discussion

In Table 2, we can see that with the increase in malware image size, the accuracy, parameters, and prediction time also increase. The malware image size will have an effect on the feature extraction ability of the MFFC. The larger the image size, the better the effect of feature extraction. But with the increase in image size to a threshold, there is little improvement in accuracy. The parameters are also increasing with the increment in malware image size. Although the parameters of image size from 128 × 128 to 256 × 256 increased to nearly 0.6 M, the accuracy improved from 97.43% to 98.72% for a total increase of 1.23%, and the increase in prediction is just only 0.63 ms. In malware classification, accuracy and prediction are more important to the parameters. We believe that it is worth to improve the accuracy at the cost of the parameters increasing in this part. Thus, the malware image size of 256 × 256 is a better choice for MFFC.

Through the observation of the result after training, we get a model that converges fast and has good generalization ability. The overall malware classification by our method for 25 malware families obtains a satisfactory performance. However, as shown in the confusion matrix, there is a major source of misclassifications, which the MFFC has difficulty in classifying samples that are variants of the same family, such as C2LOP.P and C2LOP.gen!g, Swizzor.gen!E, and Swizzor.gen!I. In other words, our model is capable of detecting variants of malware families. There is no denying that the MFFC algorithm keeps an excellent performance.

Table 3 shows that MFFC is better than the existing malware classification methods in recent years except IMCFN. The performance of the MFFC algorithm and IMCFN is similar. Our method has the same performance in precision with IMCFN but in accuracy is 0.1 lower than IMCFN. This is probably because IMCFN converts the malware binaries into malware color images while that in our method are grayscale images. The color images have more details, which are helpful in extracting features of malware images. That is a good topic for our future research. In addition, the total number of parameters in IMCFN is nearly 138 million while that in MFFC is only 1,104,041. It demonstrates that MFFC has an advantage in parameters.

Overall, our method obtains an excellent performance, which has high accuracy and high speed of prediction time, while has the ability to detect variants of malware families. Both machine learning and deep learning have excellent effects on malware detection by image classification. With the dramatically increasing number of malwares, more effective methods are in urgent need.

## 6. Conclusions

This study proposed a novel method, which is named MFFC, based on multiscale feature fusion of malware grayscale images by malware visualization, for improving the performance of malware classification and the ability of detecting malware variants. The experimental results on 25 malware families, which include 9,342 grayscale images, showed that our method keeps an excellent performance with achieving 98.72% accuracy and a good detection speed of 0.00534 seconds.

In the experiment, we found that in some malware images after size normalization will appear the change in image texture features that limited the performance of our model. This is because the original length-width ratio of the malware image is different. When we resize the malware images, the images are partially stretched resulting in image distortion. In future studies, we would like to look for a new method to realize malware image size normalization that can keep the malware image features unchanged. The transformation of malware into color images is proved to have a more excellent performance. We will improve our method by visualizing malicious codes to color images. In addition, the optimization of model hyperparameters often depends on human experience without a theoretical basis. Some of the most representative computational intelligence algorithms would be effective to solve the problem, like monarch butterfly optimization (MBO), earthworm optimization algorithm (EWA), elephant herding optimization (EHO), moth search (MS) algorithm, slime mould algorithm (SMA), and Harris hawks optimization (HHO).

## Figures and Tables

**Table 1:** Image width setting of different file sizes.

File size	Image width	File size	Image width

<10 KB	32	100 KB∼200 KB	384
10 KB∼30 KB	64	200 KB∼500 KB	512
30 KB∼60 KB	128	500 KB∼1000 KB	768
60 KB∼100 KB	256	>1000 KB	1024

**Table 2:** Performance of different input shapes of malware images.

Input shape	Accuracy (%)	Params	Prediction time (ms)

32 × 32	83.74	297,641	4.28
64 × 64	94.22	336,041	4.29
128 × 128	97.43	489,641	4.71
256 × 256	98.72	1,104,041	5.34

**Table 3:** Comparative summary of MFFC algorithm with previous malware classification algorithms.

Method	Year	Technique	Accuracy (%)	Precision (%)	Recall (%)	F1 score (%)

Nataraj et al. [6]	2011	ML	97.18	—	—	—
SPAM-GIST [10]	2016	ML	97.40	—	—	—
DL + SVM [17]	2017	DL + ML	84.92	—	—	—
Vgg-verydeep-19 [16]	2017	DL	97.32	—	—	—
GIST + SVM [18]	2018	ML	92.20	92.50	91.40	—
GIST + KNN [18]	2018	ML	91.90	92.10	91.70	—
GLCM + SVM [18]	2018	ML	93.20	93.40	93.00	—
GLCM + KNN [18]	2018	ML	92.50	92.70	92.30	—
DRBA + CNNs [18]	2018	DL	94.50	96.60	88.40	—
LGMP + KNN [11]	2019	ML	98.40	—	98.20	—
NSGA-II + CNNs [19]	2019	DL	97.60	97.60	88.40	—
Venkatraman [22]	2019	DL	96.30	91.80	91.50	91.60
Gibert et al. [28]	2019	DL	98.50	98.00	98.00	98.00
IMCFN [29]	2020	DL	98.82	98.85	98.81	98.75
DEAM-DenseNet [30]	2021	DL	98.50	96.90	96.60	96.70
**MFFC**	**2021**	**DL**	**98.72**	**98.86**	**98.72**	**98.73**

ML: machine learning; DL: deep learning.

## Data Availability

Previously reported data were used to support this study and are available at 10.1145/2016904.2016908. These prior studies (and datasets) are cited at relevant places within the text as references [6].
